# Inhibitory Action of Antidepressants on Mouse Betaine/GABA Transporter (BGT1) Heterologously Expressed in Cell Cultures

**DOI:** 10.3390/ijms13032578

**Published:** 2012-02-24

**Authors:** Gerile, Chiharu Sogawa, Kazumi Ohyama, Takashi Masuko, Tadashi Kusama, Katsuya Morita, Norio Sogawa, Shigeo Kitayama

**Affiliations:** 1 Department of Dental Pharmacology, Okayama University Graduate School of Medicine, Dentistry and Pharmaceutical Sciences, Okayama 700-8525, Japan; E-Mails: gms421006@s.okayama-u.ac.jp (G.); caoki@md.okayama-u.ac.jp (C.S.); sogawa@md.okayama-u.ac.jp (N.S.); 2 RI Research Center, Okayama University Dental School, Okayama 700-8525, Japan; E-Mail: kohyama@md.okayama-u.ac.jp; 3 Laboratory of Physiology and Anatomy, College of Pharmacy, Nihon University, Funabashi 274-8555, Japan; E-Mails: masuko.takashi@nihon-u.ac.jp (T.M.); kusama.tadashi@nihon-u.ac.jp (T.K.); 4 Department of Dental Pharmacology, Hiroshima University Graduate School of Biomedical Sciences, Hiroshima 734-8553, Japan; E-Mail: kmorita@hiroshima-u.ac.jp

**Keywords:** betaine, GABA, transporter, antidepressant, uptake inhibitor

## Abstract

Betaine/γ-aminobutyric acid (GABA) transporter (BGT1, SLC6A12) is a member of the Na^+^- and Cl^−^-dependent neurotransmitter transporter gene family with a homology to the GABA transporters (GATs), GAT1 (SLC6A1), GAT2 (SLC6A13) and GAT3 (SLC6A11) (HUGO nomenclature). Since antidepressants have been reported to inhibit GABA uptake, we examined those effects on mouse BGT1 (mBGT1) in comparison with other mouse GAT (mGAT) subtypes in the heterologously expressed cell cultures. All antidepressants tested here inhibited the [^3^H]GABA uptake through mBGT1 and mGATs in a rank order of potency with mBGT1 > mGAT1-3. Kinetic analyses for maprotilline, mianserine and trimipramine revealed that they inhibited mBGT1 and mGAT1 noncompetitively, except that mianserine competitively inhibited mBGT1. These results provided a clue to investigate the structure-function relationship of mBGT1 using antidepressants as a tool, leading to the identification of potential candidates for selective and specific inhibitors of mBGT1.

## 1. Introduction

Uptake of neurotransmitters through the plasma membrane transporter is a primary mechanism regulating extracellular neurotransmitter concentrations, thereby play a key role in the control of synaptic neurotransmission [1]. Betaine/γ-aminobutyric acid (GABA) transporter (BGT1, SLC6A12) [2,3] is a member of the Na^+^- and Cl^−^-dependent neurotransmitter transporter gene family (SLC6) with a high homology to the GABA transporters (GATs), GAT1 (SLC6A1), GAT2 (SLC6A13) and GAT3 (SLC6A11) [4]. They have 12 hydrophobic transmembrane domains (TMDs), large extracellular loop between TMD 3 and 4 with multiple glycosylation sites, and intracellular N- and C-termini [5].

Since GABA is the major inhibitory neurotransmitter in the mammalian central nervous system, GATs could be an attractive target for therapy of CNS disorders associated with GABAergic system, such as epilepsy and neuropathic pain [6,7]. Recent observations demonstrated a functional role of mouse BGT (mBGT1) or mGAT2 [8] in the control of neuronal excitability and suggested a possible utility of BGT1-selective inhibitors for the treatment of epilepsy [9]. However, in contrast to the known selective ligands for GAT1, for example, tiagabine known as a drug used clinically for treatment of partial seizure of epilepsy, no selective ligands for the other three GAT subtypes have been reported so far.

Tricyclic antidepressants (TCAs), known as the inhibitors of monoamine neurotransmitter transporters, especially transporters for serotonin (SERT) and noradrenaline (NET) [10], are therapeutically useful ligands for the treatment of neuropathic pain [11]. It seems likely that they act on inhibitory serotonergic and noradrenergic descending pathways in the spinal cord, resulting in the inhibition of pain. Since TCAs have been known to exert inhibitory effects on various targets including channels and transporters, which might be involved in neuropathic pain [12], it would be useful to evaluate a potency of TCAs in inhibiting GABA uptake through each GAT subtype including BGT1. To support this idea, Nakashita *et al*. (1997) [13] reported the inhibitory effects of various antidepressants on the three subtypes of rat GAT (GAT1-GAT3). However, there is no report of the effect of antidepressants on BGT1.

Recent success of X-ray crystallography of leucine transporter (LeuT), a bacterial homolog of mammalian Na^+^- and Cl^−^-dependent neurotransmitter transporter [14], followed by that with tricyclic antidepressant (TCA) [15,16] demonstrated the molecular map of substrate- and TCA-binding sites. Based on these findings, attempts have been made to develop a homology model of the mammalian neurotransmitter transporters including GATs [17–19]. Furthermore, recent observation by Cherubino *et al*. (2009) [20] demonstrated that K448 of rat GAT1, a counterpart of LeuT D401 composed of TCA-binding pocket, is involved in the interaction of TCA with different potencies. Since there are several differences in the putative residues involved in the interaction of TCA between GAT1 and BGT1, it was of interest to investigate the effects of TCA on BGT1 in comparison with other GAT family.

In the present study, we addressed this issue by analyzing the potency of various TCA in inhibiting mBGT1 in comparison with other GAT subtypes using [^3^H]GABA uptake assays in the cell cultures heterologously expressing mouse GAT subtypes. Further, we selected three atypical TCAs to evaluate the mode of inhibition by examining the kinetics of transport.

## 2. Results

### 2.1. Establishment of the GAT-Expressing Cell Lines

To analyze the effects of inhibitor candidates including antidepressants on the uptake of [^3^H]GABA, we established cell lines stably expressing GATs using CHO cells. Those were designated CHO/mGAT1, CHO/mGAT2, CHO/mGAT3 and CHO/mBGT1. Nomenclature of GAT subtypes accords to HUGO. Mouse GAT2 designated in the first report [8] is the counterpart of human BGT1. Therefore, in this paper we designated mBGT1 instead of mGAT2 according to the HUGO nomenclature. Similarly, mGAT2 in this paper corresponds to human GAT2 and mouse GAT3 in the first report [8], and mGAT3 in this report to human GAT3, rat GAT-B and mouse GAT4 in the first report [8]. See Supplementary Table S1 for details. The characteristics of these cell lines according to the sensitivity to inhibitors known as selective ligands to each subtypes (Supplementary Table S1) were in good agreement to those observed in the literatures, e.g., Kvist *et al*. 2009 [21] (Supplementary Table S2).

### 2.2. Comparison of the Inhibitory Potency of Antidepressants

Using these cell lines together with CHO cells stably expressing rat SERT [22,23] as a control, we analyzed the effects of 11 antidepressants on the uptake of [^3^H]GABA or [^3^H]5-HT. They inhibited [^3^H]GABA uptake by the CHO cells expressing mGAT1, mGAT2, mGAT3 and mBGT1 in a concentration-dependent fashion, as well as [^3^H]5-HT uptake through rSERT (Supplementary Figure S1). All the antidepressants tested revealed weak potency in inhibiting [^3^H]GABA uptake through GAT subtypes, as compared to SERT inhibition (Table 1). To our interest, however, these antidepressants showed higher potency in inhibiting [^3^H]GABA uptake through mBGT1 than those through other GAT subtypes, such as mGAT1, mGAT2 and mGAT3. In addition, most of them had a higher potency in inhibiting mGAT2 or mGAT3 than mGAT1.

According to the correlation analyses, potency of TCAs tested in inhibiting mBGT1 as well as mGATs correlated well with that in inhibiting rSERT, except maprotiline, mianserine and trimipramine, which revealed weak potency to inhibit SERT (Supplementary Figure S2). In addition, correlation analyses between mBGT1 and mGAT1 showed that although all these drugs displayed a greater potency in inhibiting mBGT1 than mGAT1 as mentioned above, maprotiline had a relatively lower ratio of the IC_50_ value for mGAT1 *vs*. mBGT1, while trimipramine had a greater ratio. Based on these results, we chose these three antidepressants to further analyze those effects on mBGT1 in comparison with mGAT1.

### 2.3. Kinetic Analyses of the Effects of Antidepressants on BGT1

**Figure 1:**
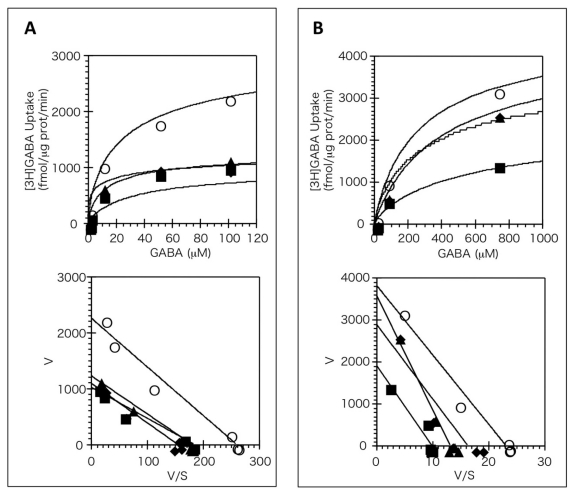
Kinetic analysis of the effects of antidepressants on the [^3^H]GABA uptake in COS cells transiently expressing mouse (m)GAT1 (**A**) and mBGT1 (**B**). Upper panels in A and B represent saturation curves, and lower panels represent Eadie-Hofstee plots. Cells were incubated with 20 nM [^3^H]GABA in the absence (open circle) or presence of maprotilline (square, 100 μM for mGAT1 and 60 μM for mBGT1), mianserine (rhombus, 200 μM for mGAT1 and 50 μM for mBGT1), and trimipramine (triangle, 100 μM for mGAT1 and 20 μM for mBGT1). Nonspecific uptake was determined in the presence of 10 mM GABA. The data represent a typical result from single experiment performed in duplicate, followed by three additional experiments with similar results.

Since all the antidepressants are known to inhibit monoamine transporter competitively [10], we analyzed the effects of the antidepressants selected on the kinetics of [^3^H]GABA uptake through mGAT1 and mBGT1 using the saturation curves and Eadie-Hofstee plots (Figure 1). Maprotiline, mianserine and trimipramine all inhibited [^3^H]GABA uptake through mGAT1 by decreasing *V*_max_ without changing *K*_m_, indicating a non-competitive manner (Figure 1 and Table 2). These compounds also inhibited mBGT1 noncompetitively, except that mianserine inhibited mBGT1 by increasing *K*_m_ without changing *V*_max_, indicating a competitive manner (Figure 1 and Table 2).

## 3. Discussion

BGT1 (SLC6A12) is a member of the Na^+^- and Cl^−^-dependent neurotransmitter transporter gene family with a high homology to the GATs, GAT1 (SLC6A1), GAT2 (SLC6A13) and GAT3 (SLC6A11) (HUGO nomenclature), and reveals GABA transport activity. However, role of BGT1 in the brain remains obscure. Since TCAs have been reported to inhibit GABA uptake [13], we examined those effects on mBGT1 in comparison with other mouse GAT subtypes in the heterologous expression systems.

The present results confirmed the previous observations demonstrating the inhibition of GATs by TCAs [13], and extend those effects on BGT1. All of the drugs tested revealed a weaker potency in inhibiting GABA uptake through the GATs and BGT1 than that in inhibiting 5-HT uptake through SERT. However, they have a greater potency in inhibiting BGT1 than GAT1-3. Furthermore, kinetic analyses revealed that trimipramine, maprotilline and mianserine inhibited BGT1 and GAT1 noncompetitively, except that mianserine competitively inhibited BGT1. Although high concentrations of TCAs necessary for inhibiting GATs in the present in vitro study are of little clinical significance, these results provided a clue to investigate the structure-function relationship of BGT1 using antidepressants, leading to the identification of potential candidates for selective and specific interaction between ligands and BGT1.

There are several differences between the results observed by Nakashita *et al*. (1997) [13] and those here regarding the potency of antidepressants in inhibiting GAT1-3. For example, they reported similar potency of amitriptyline, desipramine and maprotiline in inhibiting GAT1 and GAT3 [13], whereas we observed that they revealed a more pronounced inhibition of GAT3 than GAT1. The possible explanation for these differences may be due to the differences of cell cultures used for transfection, methods for transfection such as transient or stable transfection, or treatment with antidepressants such as simultaneous application of drugs with substrate or pretreatment. Among these, the method for drug treatment seems likely to explain such differences of the results obtained, since the dissociation rate of drugs is critical for their inhibitory potency, as suggested [10,24].

Another possibility is the difference of GATs used, such as Nakashita used rat GATs while we used mouse GATs. Amino acid sequences of these GAT subtypes display high homology between mouse and rat. Recent success of X-ray crystallography of leucine transporter (LeuT), a bacterial homolog of mammalian Na^+^- and Cl^−^-dependent neurotransmitter transporter [14], and that with TCA [15,16] demonstrated the molecular map of TCA binding sites, which consist of extracellular vestibule. However, these candidate amino acids of rat and mouse GAT subtypes are same. Therefore, given that the structural difference between rat and mouse GAT proteins results in the different sensitivity to TCA, amino acid differences in the region other than extracellular vestibule might be involved in the TCA binding site or influence the structural diversity of extracellular vestibule. Species-scanning mutagenesis of the SERT was found to reveal residues essential in selective and high-affinity recognition of antidepressants [25,26]. A restricted region in or near TMD12 has been suggested to be involved in both substrate and antagonist recognition [25], and F586 of human SERT was identified as being responsible for high affinity interactions of TCA [26]. mGAT1 shows same amino acid sequence as rGAT except W550 of mGAT1 (accession number BC059080) located in the middle of TMD12, which corresponds to G550 of rGAT1 (M59742). Therefore, this residue might be an attractive candidate to explore its importance for recognition of TCA.

In addition, the present results suggest the candidate amino acids interacting with TCA, which may result in the different sensitivity to TCA between mBGT1 and mGAT1. There are three different parts involved in the interaction with substrates (central and second substrate binding sites) and antidepressants (extracellular vestibule), as indicated previously [17–19]. K448 of mGAT1 and Q463 of mBGT1 corresponding to D401 of LeuT, which was suggested to be located in extracellular vestibule for TCA binding, is a most attractive candidate for possible sites involved in TCA interaction, since it has been reported that substitution of K448 of rat GAT1 to glutamate or aspartate revealed higher sensitivity to desipramine using oocyte expression system [20]. Therefore, it is speculated that Q463 of mBGT1 produces higher sensitivity of mBGT1 to TCA than mGAT1. In addition, I67, A310, Q313 and C413 of mBGT1 are also interesting candidates, which counterparts are A61, G297, L300 and T400 of mGAT1, A22, S256, F259 and I359 of LeuT, respectively (Supplementary Figure S3). These were suggested to be located in the central substrate-binding site, indicating the possibility that these residues are responsible to the difference of the sensitivity not only to the substrate transport but also to the TCA interaction. Since mianserine revealed a competitive inhibition of mBGT1 but noncompetitive inhibition of mGAT1, it is of interest to investigate the role of these residues in interacting with TCA.

Since it is suggested that BGT1 plays a role in regulating GABAergic activity in CNS, development of selective and specific inhibitor of BGT1 has a clinical importance [6,7,9]. BGT1 has been shown to localize in astrocytes distant from GABAergic synapses, suggesting the different role of the regulation of GABAergic transmission from that of GAT1 or GAT3, which are located in nerve endings or astrocytes neighboring synapses [4]. Recent observation demonstrated the involvement of BGT1 in regulation of epilepsy by using combination of GAT1 selective inhibitor and nonselective inhibitor [7]. However, recent studies using BGT1 knockout mice provided evidence against the previous work, which cast doubt on whether brain BGT1 plays a significant role [27]. Furthermore, BGT1 was found predominantly in liver, although its role in the liver was uncertain [28]. Nevertheless, selective and specific inhibitor of BGT1 could warrant its usefulness for clarifying these issues. The present results providing a clue to investigate the structure function relationship of BGT1 might be extended to explore such ligands.

## 4. Experimental Section

### 4.1. Materials

Drugs used were: cocaine hydrochloride (Takeda Chemical Industries, Ltd., Osaka, Japan), 5-HT, pargyline hydrochloride (Nakalai Tesque, Kyoto, Japan), paroxetine (Tronto Research Chemicals Inc., North York, ON, Canada), amitriptyline, amoxapine, clomipramine, maprotiline, mianserine, nortriptyline, doxepin, protriptyline, trimipramine, zimelidine (Sigma-Aldrich Co., St. Louis, MO, USA), desipramine, imipramine, (Wako Pure Chemical Industry Co. Ltd., Tokyo, Japan). RivatraAce, KOD-plus DNA polymerase from TOYOBO (Tokyo, Japan), restriction emzymes from New England Biolabs (Beverly, MA, USA), DNA purification kits from Qiagen (Hilden, Germany), transfection reagent FuGENE6 from Roche Diagnostics (Mannheim, Germany), reagents for cell culture from Sigma and Invitrogen (Carlsbad, CA, USA).

[^3^H]GABA (0.87 TBq/mmol) and [^3^H]5-HT (1.11 TBq/mmol) were purchased from NEN Life Science Products, Inc. (Boston, MA, USA).

### 4.2. cDNA Constructs

mGAT1 cDNA(4.2 kb) in pBluescript II has been cloned previously [29], and its EcoRI/ApaI fragment (2.2 kb) containing open reading frame of mGAT1 was subcloned into pcDNA3.

mGAT2 (clone ID; C730037J22) and mGAT3 (clone ID; B730043P22) cDNAs from RIKEN Mouse FANTOM Clones were obtained through KK DANAFORM (Yokohama, Japan) [30]. SfiI fragment of mGAT2 cDNA was polished and subcloned initially into pBluescript II SK- at EcoRV site, and then NotI/SalI fragment was subcloned into pcDNA3 at NotI/XhoI site. mGAT3 cDNA was subcloned into pcDNA3 at EcoRI/XbaI site.

mBGT1 was purchased from Origene Technologies, Inc. (Rockville, MD, USA), and subcloned into pcDNA3 at KpnI/XbaI site.

Rat SERT (rSERT) cDNA used as a control has been cloned previously [22].

### 4.3. Cell Culture and Expression

COS cells were maintained in high glucose Dulbecco’s modified Eagle’s medium (DMEM) supplemented with 10% fetal calf serum, 100 units/mL penicillin-G, 100 μg/mL streptomycin and 2.5 μg/mL fungizone at 37 °C under 5% CO_2_/95% air. CHO cells were cultivated in alpha-minimum essential medium (α-MEM), supplemented with 10% fetal calf serum, 100 units/mL penicillin-G, 100 μg/mL streptomycin and 2.5 μg/mL fungizone at 37 °C under 5% CO_2_/95% air.

For uptake assays in cell lines stably expressing mouse GATs, CHO cells were transfected with cDNA using FuGENE6 transfection reagent according to the manufacturer’s directions, and subsequently cultured in the medium containing G418 to select positive clones. CHO cells stably expressing rat SERT have been made previously [23].

For transient expression experiments, COS-7 cells at subconfluence were harvested and transfected with cDNA of GATs by electroporation [31]. A parallel transfection with the pcDNA3 vector alone was performed each time as a negative control. After the electroporation, cells were diluted in the culture medium, plated in 24- or 48-well culture plates, and cultured for 2 days.

### 4.4. Uptake Assay

Cells were washed three times with oxygenated Krebs Ringer HEPES-buffered solution (KRH; 125 mM NaCl, 5.2 mM KCl, 1.2 mM CaCl_2_, 1.4 mM MgSO_4_, 1.2 mM KH_2_PO_4_, 5 mM glucose, and 20 mM HEPES, pH 7.3) and incubated for 10 min at 37 °C with 10 nM [^3^H]GABA or other radio-labeled ligands, as described previously [31,32]. Aminooxyacetic acid (100 μM) and ascorbic acid (100 μM) plus pargyline (50 μM) were added to the incubation solution during GABA and 5-HT uptake assays, respectively. After the removal of excess radioligands by aspiration, the cells were washed three times rapidly with ice-cold KRH and any radioactivity remaining in the cells was extracted with NaOH, and measured by liquid scintillation spectrometry. Nonspecific uptake was determined in mock-transfected cells and also in each plate in the presence of 10mM cold GABA for GABA uptake or 10 μM cocaine for 5-HT uptake. For the kinetic analysis, cells were incubated for 8 min at 37 °C in KRH containing 20 nM [^3^H]GABA and 0.1–100 μM cold GABA. To exclude the [^3^H]GABA uptake through endogenous GAT in COS-7 cells, 100 μM β-alanine was added to the incubation solution for the kinetic analysis of mBGT1.

### 4.5. Statistical Analysis

Data were analyzed by Eadie-Hofstee plots using Prism 5 (GraphPad Software, Inc., San Diego, CA, USA). Statistical analyses were performed using the analysis of variance (ANOVA) with pairwise comparison by the Scheffe method and the Student’s *t*-test.

## 5. Conclusions

All antidepressants tested here inhibited the [^3^H]GABA uptake through mBGT1 and mGATs in a rank order of potency with mBGT1 > mGAT1-3. Kinetic analyses for maprotilline, mianserine and trimipramine revealed that they inhibited mBGT1 and mGAT1 noncompetitively, except that mianserine competitively inhibited mBGT1. Based on the LeuT structure, the results from the present study focused several residues responsible for the difference of the sensitivity not only to the substrate transport but also to the antidepressant interaction. The finding that mianserine revealed a competitive inhibition of mBGT1 but noncompetitive inhibition of mGAT1 provided a clue to investigate the structure-function relationship of BGT1, which might lead to the development of potential candidates for selective and specific BGT1 inhibitors.

## Supplementary Information



## Figures and Tables

**Table 1:** Comparison of the inhibitory action of antidepressants on mouse γ-aminobutyric acid (GABA) transporter subtypes and rat serotonin transporter (SERT). CHO cells stably expressing mouse GABA transporter (GAT) subtypes and rat SERT were incubated with 10 nM [^3^H]GABA or 10 nM [^3^H]5-HT for 10 min in the absence or presence of various concentrations of antidepressants tested. Values for mGAT1 and mBGT1 represent the mean ± SEM for three–four experiments each performed in triplicate, and the data were analyzed statistically.

Drugs	IC_50_ (μM)				
	
	mBGT1	mGAT1	mGAT2	mGAT3	rSERT
Amitriptyline	33.0 ± 9.9 *	100.6 ± 11.7	45.8	61.5	0.14
Amoxapine	78.3 ± 9.5 *	287.9 ± 80.5	134.6	195.4	1.62
Clomipramine	27.2 ± 6.1	93.3 ± 39.8	41.8	40.2	0.05
Desipramine	75.3 ± 6.3 *	268.4 ± 36.7	122.5	80.3	1.23
Doxepine	59.9 ± 11.2 **	222.1 ± 14.7	129.2	115.5	0.58
Imipramine	44.5 ± 2.2 *	222.5 ± 58.5	105.9	108.1	0.75
Maprotilline	61.3 ± 2.1 **	123.5 ± 8.4	98.0	106.1	27.5
Mianserine	57.4 ± 3.9 **	221.6 ± 9.9	131.1	91.0	7.63
Nortriptyline	47.0 ± 2.1 *	135.7 ± 21.9	87.1	74.1	0.63
Protriptyline	43.6 ± 10.2 **	166.8 ± 22.1	77.2	113.0	0.91
Trimipramine	21.8 ± 3.8 *	181.4 ± 72.9	85.6	64.6	5.89

* *P* < 0.05,

** *P* < 0.01 *vs*. mGAT1.

Data for mGAT2, mGAT3, and rSERT represent IC_50_ values from single experiment or the mean values from two experiments.

**Table 2:** Changes in the GABA transport kinetics induced by antidepressants in COS-7 cells expressing mGAT1 and mBGT1. Cells were incubated with 20 nM [^3^H]GABA in the absence (open circle) or presence of maprotilline (100 μM for mGAT1 and 60 μM for mBGT1), mianserine (200 μM for mGAT1 and 20 μM for mBGT1), and trimipramine (100 μM for mGAT1 and 20 μM for mBGT1). Nonspecific uptake was determined in the presence of 10 mM GABA. Values represent mean ± SEM of four experiments each performed in duplicate.

Transporter	Drugs	*K*_m_ (μM)	*V*_max_ (ratio to control #)
***mGAT1***	Control	13.04 ± 1.87	1.00
	Maprotiline	9.38 ± 2.44	0.48 ± 0.04 *
	Mianserine	9.93 ± 2.67	0.47 ± 0.04 *
	Trimipramine	9.33 ± 2.39	0.37 ± 0.05 *

***mBGT1***	Control	202.2 ± 34.8	1.00
	Maprotiline	242.7 ± 56.2	0.47 ± 0.06 *
	Mianserine	305.2 ± 56.4 *	0.90 ± 0.03
	Trimipramine	254.9 ± 34.4	0.86 ± 0.06

* *P* < 0.05 *vs*. Control.

# *V*_max_ values were calculated as ratio to control in each experiment, and analyzed statistically.

*V*_max_ values of controls for mGAT1 and mBGT1 were 2772.2 ± 1551.0 and 4007.5 ± 897.5 fmol/μg protein/min, respectively.

## References

[1] Iversen L.L. (1971). Role of transmitter uptake mechanisms in synaptic neurotransmission. Br. J. Pharmacol.

[2] Yamauchi A., Uchida S., Kwon H.M., Preston A.S., Robey R.B., Garcia-Perez A., Burg M.B., Handler J.S. (1992). Cloning of a Na^+^- and Cl^−^-dependent betaine transporter that is regulated by hypertonicity. J. Biol. Chem.

[3] Borden L.A., Smith K.E., Gustafson E.L., Branchek T.A., Weinshank R.L. (1995). Cloning and expression of a betaine/GABA transporter from human brain. J. Neurochem.

[4] Borden L.A. (1996). GABA transporter heterogeneity: Pharmacology and cellular localization. Neurochem. Int.

[5] Amara S.G., Kuhar M.J. (1993). Neurotransmitter transporters: Recent progress. Ann. Rev. Neurosci.

[6] Dalby N.O. (2003). Inhibition of γ-aminobutyric acid uptake: Anatomy, physiology and effects against epileptic seizures. Eur. J. Pharmacol.

[7] Madsen K.K., White H.S., Schousboe A. (2010). Neuronal and non-neuronal GABA transporters as targets for antiepileptic drugs. Pharmacol. Ther.

[8] Liu Q.-R., Lopez-Corcuera B., Mandiyan S., Nelson H., Nelson N. (1993). Molecular characterization of four pharmacologically distinct γ-aminobutyric acid transporters in mouse brain. J. Biol. Chem.

[9] Schousboe A., Larsson O.M., Sarup A., White H.S. (2004). Role of the betaine/GABA transporter (BGT-1/GAT2) for the control of epilepsy. Eur. J. Pharmacol.

[10] Langer Z.S., Schoemaker H. (1988). Effects of antidepressants on monoamine transporters. Prog. Neuropsychopharmacol. Biol. Psychiat.

[11] Todd A.J. (2010). Neuronal circuitry for pain processing in the dorsal horn. Nat. Rev. Neurosci.

[12] Mico J.A., Ardid D., Berrocoso E., Eschalier A. (2006). Antidepressants and pain. Trends Pharmacol. Sci.

[13] Nakashita M., Sasaki K., Sakai N., Saito N. (1997). Effects of tricyclic and tetracyclic antidepressants on the three types of GABA transporter. Neurosci. Res.

[14] Yamashita A., Singh S.K., Kawate T., Jin Y., Gouaux E. (2005). Crystal structure of a bacterial homologue of Na^+^/Cl^−^ -dependent neurotransmitter transporters. Nature.

[15] Singh S.K., Yamashita A., Gouaux E. (2007). Antidepressant binding site in a bacterial homolog of neurotransmitter transporters. Nature.

[16] Zhou Z., Zhen J., Karpowich N.K., Goetz R.M., Law C.J., Reith M.E.A., Wang D.N. (2007). LeuT-desipramine structure reveals how antidepressants block neurotransmitter reuptake. Science.

[17] Nyola A., Karpowich N.K., Zhen J., Marden J., Reith M.E., Wang D.-N. (2010). Substrate and drug binding sites in LeuT. Curr. Opin. Struct. Biol.

[18] Skovstrup S., Taboureau O., Brauner-Osborne H., Jorgensen F.S. (2010). Homology modeling of the GABA transporter and analysis of tiagabine binding. Chem. Med. Chem.

[19] Kardos J., Pallo A., Bencsura A., Simon A. (2010). Assessing structure, function and druggability of major inhibitory neurotransmitter γ-aminobutyrate symporter subtypes. Curr. Med. Chem.

[20] Cherubino F., Miszner A., Renna M.D., Sangaletti R., Giovannardi S., Bossi E. (2009). GABA transporter lysine 448: A key residue for tricyclic antidepressants interaction. Cell. Mol. Life Sci.

[21] Kvist T., Christiansen B., Jensen A.A., Brauner-Osborne H. (2009). The four human γ-aminobutyric acid (GABA) transporters: Pharmacological characterization and validation of a highly efficient screening assay. Comb. Chem. High Throughput Screen.

[22] Sato T., Kitayama S., Mitsuhata C., Ikeda T., Morita K., Dohi T. (2000). Selective inhibition of monoamine neurotransmitter transporters by synthetic local anesthetics. Naunyn-Schmiedeberg’s Arch. Pharmacol.

[23] Sogawa C., Sogawa N., Tagawa J., Fujino A., Ohyama K., Asanuma M., Funada M., Kitayama S. (2007). 5-Methoxy-*N*,*N*-diisopropyltryptamine (Foxy), a selective and high affinity inhibitor of serotonin transporter. Toxicol. Lett.

[24] Rudnick G. (2007). What is an antidepressant binding site doing in a bacterial transporter?. ACS Chem. Biol.

[25] Barker E.L., Kimmel H.L., Blakely R.D. (1994). Chimeric human and rat serotonin transporters reveal domains involved in recognition of transporter ligands. Mol. Pharmacol.

[26] Barker E.L., Blakely R.D. (1996). Identification of a single amino acid, phenylalanine 586, that is responsible for high affinity interactions of tricyclic antidepressants with the human serotonin transporter. Mol. Pharmacol.

[27] Lehre A.C., Rowley N.M., Zhou Y., Holmseth S., Guo C., Holen T., Hua R., Laake P., Olofsson A.M., Poblete-Naredo I. (2011). Deletion of the betaine-GABA transporter (BGT1; slc6a12) gene does not affect seizure thresholds of adult mice. Epilepsy Res.

[28] Zhou Y., Holmseth S., Hua R., Lehre A.C., Olofsson A.M., Poblete-Naredo I., Kempson S.A., Danbolt N.C. (2012). The betaine-GABA transporter (BGT1, slc6a12) is predominantly expressed in the liver and at lower levels in the kidneys and at the brain surface. Am. J. Physiol. Renal Physiol.

[29] Gregor P., Patel A., Shimada S., Lin C.L., Rochelle J.M., Kitayama S., Seldin M.F., Uhl G.R. (1993). Murine serotonin transporter: sequence and localization to chromosome 11. Mammal. Genome.

[30] Carninci P., Kasukawa T., Katayama S., Gough J., Frith M.C., Maeda N., Oyama R., Ravasi T., Lenhard B., Wells C. (2005). The Transcriptional landscape of the mammalian genome. Science.

[31] Kitayama S., Shimada S., Xu H., Markham L., Donovan M.D., Uhl G.R. (1992). Dopamine transporter site-directed mutations differentially alter substrate transport and cocaine binding. Proc. Natl. Acad. Sci. USA.

[32] Sugimura M., Kitayama S., Morita K., Irifune M., Takarada T., Kawahara M., Dohi T. (2001). Effects of volatile and intravenous anesthetics on the uptake of GABA, glutamate and dopamine by their transporters heterologously expressed in COS cells and in rat brain synaptosomes. Toxicol. Lett.

