# Supplementary material for: Inhibitory Action of Antidepressants on Mouse Betaine/GABA Transporter (BGT1) Heterologously Expressed in Cell Cultures
Source: Int J Mol Sci. 2012 Feb 24;13(3):2578–89. doi: 10.3390/ijms13032578 (PMC3317675; doi:10.3390/ijms13032578)
Supplement: Supplementary file 1 [file ijms-13-02578-s001.pdf]

Supplementary Information

Supplementary Table S1. Classification of the GABA transporters.

		BGT1	GAT1	GAT2	GAT3
**alternate**	Human	hBGT1	hGAT1	hGAT2	hGAT3
	Mouse	mGAT2	mGAT1	mGAT4	mGAT3
	Rat		rGAT-A		rGAT-B
**gene ID**		SLC6A12	SLC6A1	SLC6A13	SLC6A11
**Accession number#**		BC019211	BC059080	AK149557	AK140423
**Substrates**		GABA, Betaine	GABA, Nipecotate	GABA, β-Alanine	GABA, β-Alanine
**Inhibitors**		NNC05-2090, EF1502	Tiagabine, SKF89976A, EF1502, NNC05-2090	SNAP-5114	SNAP-5114, NNC05-2090

#Accession number for cDNA used in the present study.

Supplementary Table S2. IC_{50} (μM) of the GABA uptake inhibitors (substrates) in inhibiting [^{3}H]GABA uptake by CHO cells stably expressing mouse GABA transporter subtypes. Cells in 48-well culture plate were incubated with 10 nM [^{3}H]GABA for 10 min in the presence or absence of inhibitors. Uptake of [^{3}H]GABA was determined in duplicate or triplicate from single experiment, and IC_{50} was calculated using Prism5. Specific uptake of [^{3}H]GABA by mGAT1, mGAT2, mGAT3 and mBGT1 in the absence of inhibitors was 8992.5, 2776.5, 2594.3 and 273.0 dpm/well, respectively.

Inhibitors	mGAT1	mGAT2	mGAT3	mBGT1
GABA	6.4	13	4.6	115
Nipecotic acid	9.7	113	86	7098
β-Alanine	>300	25	18	>1000
Betaine	>10000	>10000	>10000	693

Supplementary Figure S1. Inhibition of the GABA and serotonin transporters by antidepressants. Effects of amitryptiline (A), maprotilline (B), mianserine (C) and trimipramine (D) on uptake of [^{3}H]GABA and [^{3}H]5-HT was examined in the CHO cells stably expressing mouse GAT subtypes and rat SERT. Uptake assays were performed in 48-well plates for mGATs and 24-well plates for rSERT. Each panel shows a typical result from single experiment performed in duplicate or triplicate, and data were expressed as % of control (uptake in the absence of drugs tested). Control uptake by mGAT1, mGAT2, mGAT3, mBGT1 and rSERT was 7395.4, 2933.7, 15845.9, 466.7 and 12655.4 dpm/well, respectively.

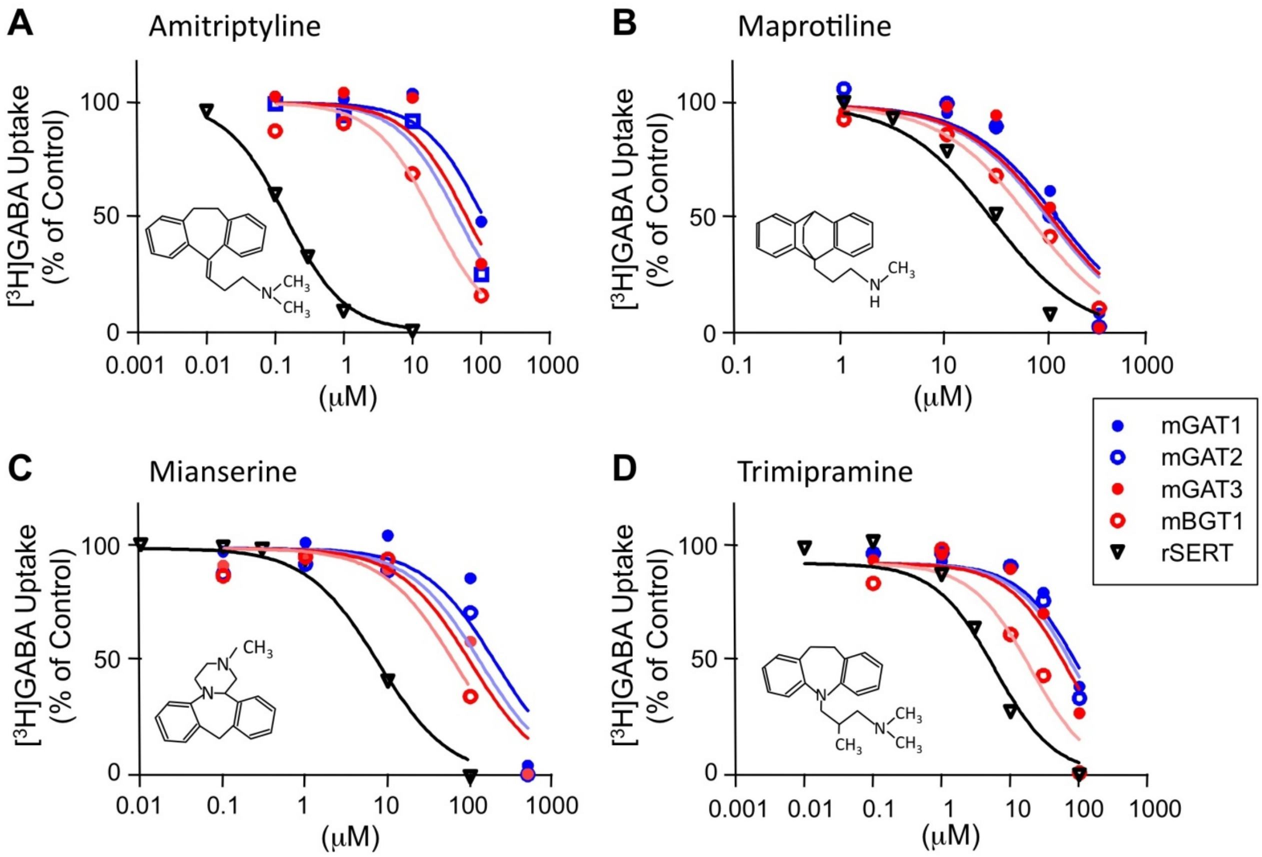

Supplementary Figure S2. Correlation analyses of the potency of antidepressants in inhibiting the GABA and serotonin transporters. IC_{50} values (μM) of the inhibitory potencies of 11 antidepressants including maprotiline (circled and numbered 1), mianserine (circled and numbered 2) and trimipramine (circled and numbered 3) were analyzed for rSERT *vs.* mGAT1 (A), rSERT *vs.* mGAT2 (B), rSERT *vs.* mGAT3 (C), rSERT *vs.* mBGT1 (D), and mGAT1 *vs.* mBGT1 (E). Values in each panel represent Pearson's correlation coefficient (r) and calculated P. r and P in panels A~D represent those calculated by excluding maprotiline, mianserine and trimipramine. When values for those antidepressants were included, r decreased and no significance was observed ($P > 0.05$). Correlation between GAT subtypes was significant even if values for those antidepressants were included. Values in *E* represent r and P when calculated by including maprotiline, mianserine and trimipramine.

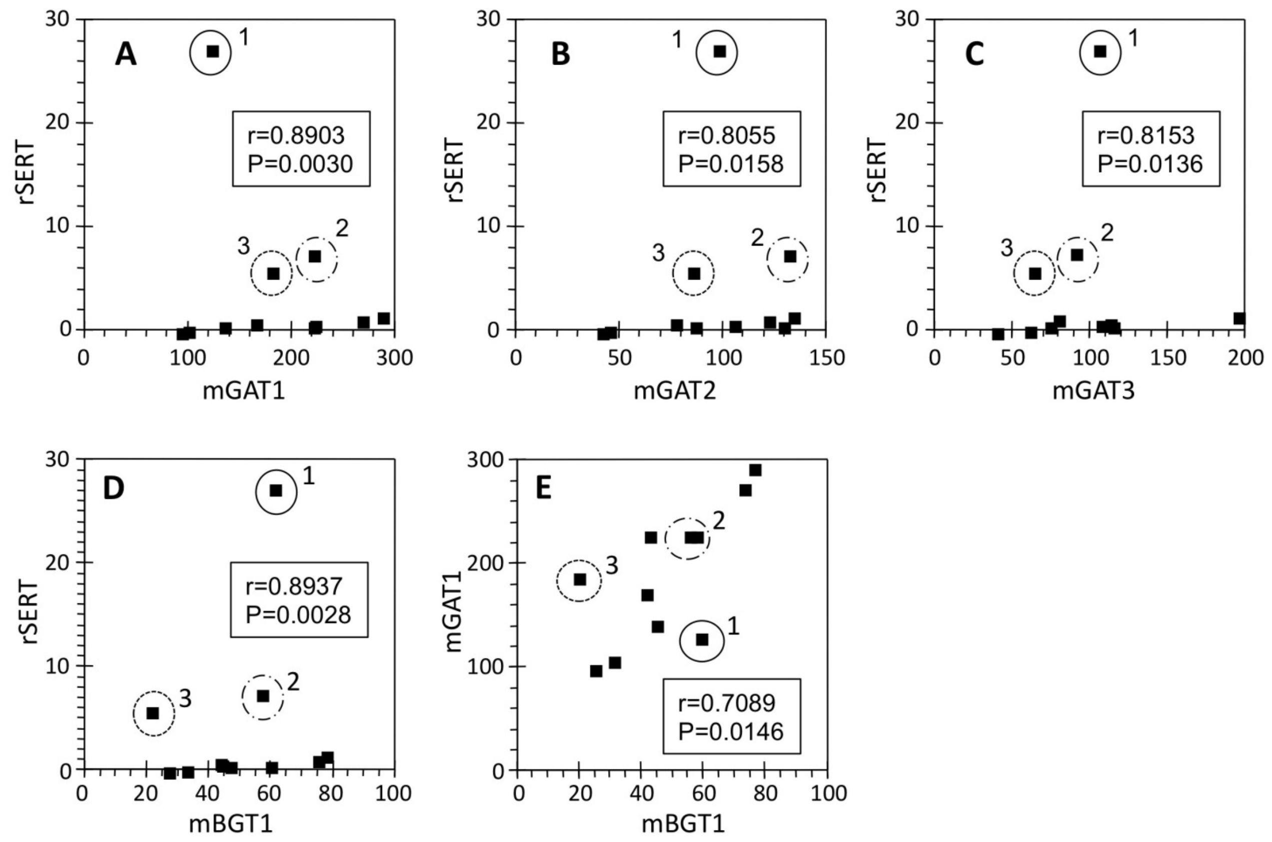

Supplementary Figure S3. Comparison of the amino acid sequences between mGATs, rat SERT and LeuT at the regions predicted for antidepressant- and substrate-binding site. Candidate amino acids for extracellular vestibule (EV) tricyclic antidepressant (TCA)-binding site, central substrate-binding site (S1) and second substrate-binding site (S2) between mGAT1, mGAT2, mGAT3, mBGT1 and rSERT are aligned with those of LeuT, a bacterial homolog of Na^+- and Cl^--dependent neurotransmitter transporter. Amino acid residues of mBGT1 different from mGAT1 were indicated in bold letter and highlighted.

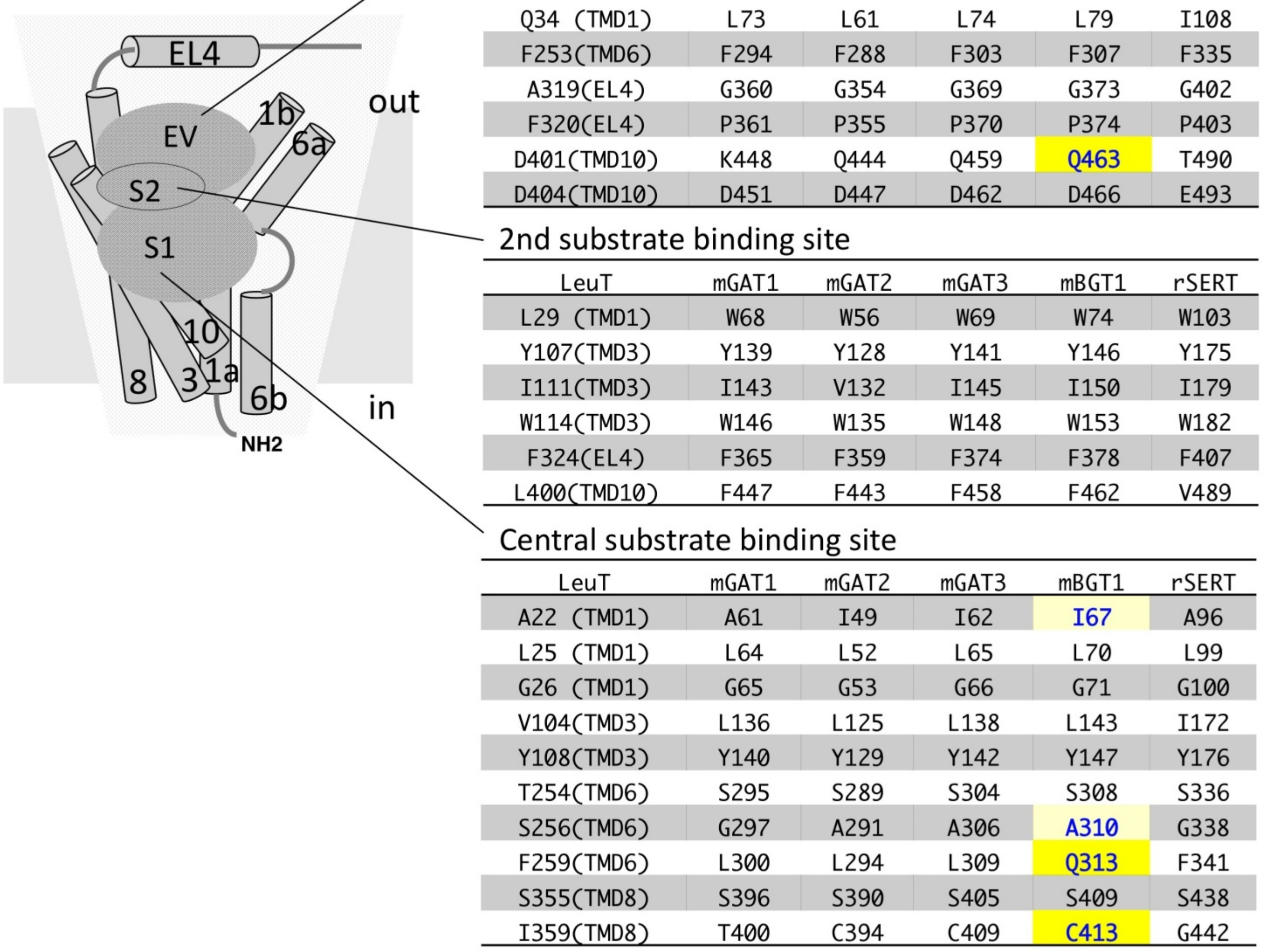

EV: Extracellular vestibule (TCA binding site)

LeuT	mGAT1	mGAT2	mGAT3	mBGT1	rSERT
R30 (TMD1)	R69	R57	R70	R75	R104
Q34 (TMD1)	L73	L61	L74	L79	I108
F253(TMD6)	F294	F288	F303	F307	F335
A319(EL4)	G360	G354	G369	G373	G402
F320(EL4)	P361	P355	P370	P374	P403
D401(TMD10)	K448	Q444	Q459	**Q463**	T490
D404(TMD10)	D451	D447	D462	D466	E493

2nd substrate binding site

LeuT	mGAT1	mGAT2	mGAT3	mBGT1	rSERT
L29 (TMD1)	W68	W56	W69	W74	W103
Y107(TMD3)	Y139	Y128	Y141	Y146	Y175
I111(TMD3)	I143	V132	I145	I150	I179
W114(TMD3)	W146	W135	W148	W153	W182
F324(EL4)	F365	F359	F374	F378	F407
L400(TMD10)	F447	F443	F458	F462	V489

Central substrate binding site

LeuT	mGAT1	mGAT2	mGAT3	mBGT1	rSERT
A22 (TMD1)	A61	I49	I62	**I67**	A96
L25 (TMD1)	L64	L52	L65	L70	L99
G26 (TMD1)	G65	G53	G66	G71	G100
V104(TMD3)	L136	L125	L138	L143	I172
Y108(TMD3)	Y140	Y129	Y142	Y147	Y176
T254(TMD6)	S295	S289	S304	S308	S336
S256(TMD6)	G297	A291	A306	**A310**	G338
F259(TMD6)	L300	L294	L309	**Q313**	F341
S355(TMD8)	S396	S390	S405	S409	S438
I359(TMD8)	T400	C394	C409	**C413**	G442

© 2012 by the authors; licensee MDPI, Basel, Switzerland. This article is an open access article distributed under the terms and conditions of the Creative Commons Attribution license (http://creativecommons.org/licenses/by/3.0/).
